# Editorial: Women with neuropsychiatric disorders: understanding & personalizing needs

**DOI:** 10.3389/fpsyt.2025.1719997

**Published:** 2025-12-01

**Authors:** Ágota Barabássy, Zsófia Borbála Dombi, Kristina Gemzell-Danielsson, Maria Judit Molnar

**Affiliations:** 1 Global Medical Division, Gedeon Richter Plc., Budapest, Hungary; 2 Department of Women’s and Children’s Health, Karolinska Institutet, Stockholm, Sweden; 3 Institute of Genomic Medicine and Rare Disorders, Semmelweis University, Budapest, Hungary

**Keywords:** women’s health, neuropsychiatric disorders, psychiatry, unmet needs, gynaecology

Neuropsychiatric disorders are clinically recognized conditions of the brain in which thoughts, perceptions, emotions, or behaviours are disrupted, significantly affecting daily functioning (1). They range from single symptoms such as anxiety or fatigue to complex illnesses like schizophrenia, bipolar disorder, or major depression.

Hormonal transitions across the female lifespan i.e., pregnancy, postpartum, perimenopause, and menopause, represent windows of heightened vulnerability (2). Even previously healthy women are at risk: postpartum depression affects up to 17% (3), and perimenopause carries a ~40% increased risk of depression compared to pre-menopause (4).

Women with pre-existing psychiatric illness face even greater challenges. In bipolar disorder, relapse rates reach 71% if mood stabilizers are stopped during pregnancy (5); maintenance therapy reduces risk by about two-thirds (6). Severe mental illness confers a ~24% chance of major relapse during pregnancy, often linked to medication discontinuation (7). These data underscore a dual vulnerability: new-onset illness in otherwise healthy women and relapse in those with established diagnoses.

During pregnancy, depression occurs in 7–13% of women (8). Nearly half who discontinue antidepressants relapse, most often in the first trimester (9). Importantly, untreated depression has significant implications as it is linked to preterm birth, low birth weight, and impaired neurodevelopment (10). Postpartum, 4–20% experience depression, often triggered by the abrupt drop in estrogen and progesterone (11). Long-term studies suggest childbirth can mark a turning point in maternal mental health, with sustained antidepressant needs (12).

The menopausal transition is another critical period. Fluctuating estrogen levels increase risks of depression, mood instability, and even psychosis (13). Within four years after menopause, major depression risk rises by 30% and mania by over 100%, supporting the “estrogen protection” hypothesis (14). Hormones may act as neuroprotective modulators, and therapies such as transdermal estradiol (15) or neuroactive steroids like brexanolone have shown benefit in perimenopausal and postpartum depression (16).

Thus, clinical management must balance maternal stability with reproductive safety. Across all phases, principles should include shared decision-making, preconception counselling, close monitoring, and interdisciplinary care. Ultimately, untreated psychiatric illness often poses greater risks than carefully managed pharmacotherapy.

## Articles of the Research Topic

The first contributions in this Research Topic focus on cariprazine, a D_2_/D_3_ receptor partial agonist. The mechanism of action of cariprazine and favourable tolerability profile make it particularly interesting for investigating novel applications beyond its current approval in women. Indeed, one study by Pappa et al. presents the first case series evaluating the efficacy and tolerability of cariprazine in female forensic inpatients with emotionally unstable personality disorder (EUPD), a population with high unmet clinical needs. Eight women were treated with cariprazine (3.0–6.0 mg) and demonstrated reductions in the mean total PANSS scores, as well as improvements in positive, negative, and general psychopathology domains. Although changes in global clinical impression were modest, the favourable side effect profile and treatment adherence highlight cariprazine’s potential as a therapeutic option in EUPD, particularly for patients with psychotic symptoms or previous intolerance to other antipsychotics.

Complementing this, a case report by Herold et al. investigates cariprazine maintenance during pregnancy in a patient with schizophrenia. Women are often excluded from clinical trials and are required to use contraception when enrolled, leaving a gap in evidence regarding the safety of psychotropic medications during pregnancy. This report shows that continuing cariprazine treatment was protective against relapses, with no adverse effects on the course of pregnancy or the newborn’s health. While based on a single patient, this finding is important, providing preliminary evidence for the safe management of schizophrenia in pregnant women, a highly vulnerable population.

A third cariprazine case by Dmuhovskis and Taube illustrates the combination of cariprazine with clozapine for the treatment of psychosis in a young woman with schizophrenia. The combination treatment resulted in marked clinical improvement, with the patient returning to work, experiencing enhanced well-being, and reporting no side effects. This example highlights the potential of cariprazine as an augmentation strategy for treatment-resistant schizophrenia in women, supporting functional recovery and quality of life.

Further, a case series of five women with zolpidem dependence (treated at the PROMUD program, a women-specific outpatient service in Brazil) demonstrates the effectiveness of a multidisciplinary approach combining group therapies, weekly psychiatric appointments, and tailored interventions. The cases underscore the importance of women-focused programs in addressing substance use, accounting for comorbidities, trauma histories, and social vulnerabilities unique to women (Leal et al.).

Finally, a study on the life dispositions of caregivers of family members with mental disorders highlights the diverse experiences and support needs of women in caregiving roles. Children caring for a parent with a mental disorder can be the most vulnerable, exhibiting lower well-being, self-esteem, and meaning in life, while parents caring for a child with mental disorders display more resilience but still benefits from informational and motivational support. These findings underscore the importance of considering women’s psychosocial context in interventions, extending beyond individual symptom management to family systems and relational dynamics (Ivanova et al.).

## Conclusions

Taken together, these contributions emphasize that women with neuropsychiatric disorders require nuanced, personalized approaches that account for biological, hormonal, and social differences across the lifespan. From reproductive considerations and pregnancy safety to caregiving responsibilities, understanding the intersection of these factors is essential. While this Research Topic does not aim to separate men and women categorically, it highlights situations where gender-sensitive strategies may optimize care and outcomes.

By integrating insights from pharmacology, psychosocial interventions, and family dynamics, this Research Topic advances understanding of women-specific needs and underscores the importance of personalized care. Future research should continue to explore gender-specific responses, ensure adequate representation of women in clinical trials, and develop guidelines that account for the complex interplay between biology, hormones, and psychosocial factors.
